# Perceptions and Attitudes Towards Artificial Intelligence Among Education and Mental Health Professionals: A Comparative Pilot Study

**DOI:** 10.1192/j.eurpsy.2025.1427

**Published:** 2025-08-26

**Authors:** A. Alberca-González, E. Fernández-Jiménez

**Affiliations:** 1 Department of Psychiatry, Clinical Psychology and Mental Health, La Paz University Hospital, Madrid; 2 Faculty of Social Sciences and Communication, Universidad Europea de Madrid, Villaviciosa de Odón; 3 Hospital La Paz Institute for Health Research (IdiPAZ), Madrid, Spain

## Abstract

**Introduction:**

The integration of Artificial Intelligence (AI) in education and mental health fields represents an innovative path to optimize diagnostic, intervention, and monitoring processes, although it poses ethical and technical challenges that must be carefully assessed. Understanding how professionals in these areas perceive this technology is essential to support its informed and effective adoption.

**Objectives:**

This study aimed to explore and compare perceptions and attitudes towards AI among professionals in education and mental health by examining key dimensions such as efficiency, personalization, diagnosis, monitoring, privacy, and validation.

**Methods:**

A cross-sectional pilot study was conducted with a sample of 17 professionals divided into two groups: five from the educational sector and 12 from the clinical-mental health sector. An online structured questionnaire, designed ad hoc, was used, comprising ten dimensions addressing diagnosis, monitoring, efficiency, data analysis, quality, ethics, dependency, validation, ease of use, and AI acceptance. All participants provided written informed consent to participate in this study.

**Results:**

The findings are as follows:

- High agreement in perceived efficiency across both sectors (>80%).
- Greater optimism in personalization within the educational sector (80% vs. 58.3% in mental health).
- General concern about the validation of AI systems (94.1% consider it insufficiently rigorous).
- Significant resistance to AI implementation (82.4%).
- Key perceived barriers: Data privacy (85%) and risk of dehumanization (76%).

**Conclusions:**

While AI’s potential to enhance efficiency is acknowledged in both areas, reservations persist concerning the validity and safety of these systems, particularly regarding data privacy and the integrity of professional-patient or student relationships. These findings suggest the need to develop comprehensive validation programs and specific protocols that facilitate the ethical and technically sound implementation of AI in these fields.

**Disclosure of Interest:**

None Declared

